# Supplementary material for: Integrating multiple genomic technologies to investigate an outbreak of carbapenemase-producing Enterobacter hormaechei
Source: Nat Commun. 2020 Jan 24;11:466. doi: 10.1038/s41467-019-14139-5 (PMC6981164; doi:10.1038/s41467-019-14139-5)
Supplement: Supplementary file 1 — Supplementary Information [file 41467_2019_14139_MOESM1_ESM.pdf]

# Supplementary Information

## Integrating multiple genomic technologies to investigate an outbreak of carbapenemase-producing *Enterobacter hormaechei*

**Roberts et al.**

# Supplementary Tables

## Supplementary Table 1: Assembly metrics

| Strain | Location | Depth[2] | Contigs[3] | Largest contig | N50 | Total Length |
|---|---|---|---|---|---|---|
| Ecl1 (JRFQ01000001) | RBWH | 30× | 83 | 514 952 | 175 645 | 5 199 581 |
| Ecl1 (reassembly) [1] | RBWH | 36× | 90 | 515 066 | 256 013 | 5 223 643 |
| MS7884 | RBWH | 93× | 84 | 556 131 | 259 415 | 5 225 529 |
| MS7885 | RBWH | 126× | 87 | 515 066 | 208 661 | 5 226 022 |
| MS7886 | RBWH | 85× | 85 | 556 628 | 259 182 | 5 228 488 |
| MS7887 | RBWH | 104× | 86 | 385 936 | 259 128 | 5 214 118 |
| MS7888 | RBWH | 99× | 86 | 406 431 | 259 182 | 5 227 406 |
| MS7889 | RBWH | 80× | 79 | 357 241 | 195 846 | 5 181 005 |
| MS7890 | RBWH | 91× | 84 | 406 431 | 208 661 | 5 195 590 |
| MS7891 | RBWH | 124× | 83 | 556 346 | 283 853 | 5 195 194 |
| MS7892 | RBWH | 121× | 89 | 388 759 | 259 518 | 5 194 114 |
| MS7893 | RBWH | 140× | 83 | 515 066 | 259 182 | 5 193 144 |
| MS7923 | Hospital A | 109× | 61 | 899 556 | 301 561 | 5 007 334 |
| MS7924 | Hospital A | 76× | 73 | 1 033 606 | 327 391 | 5 150 594 |
| MS7925 | Hospital A | 91× | 96 | 622 503 | 143 352 | 5 137 892 |
| MS7926 | Hospital B | 98× | 83 | 721 578 | 282 470 | 5 203 172 |
| MS8077 | Hospital B | 98× | 82 | 588 399 | 307 036 | 5 209 475 |
| MS8078 | Hospital B | 71× | 116 | 329 971 | 146 076 | 5 028 820 |
| MS8079 | Hospital B | 103× | 83 | 733 216 | 261 436 | 5 161 736 |

[1] Genome reassembled to try and fix erroneous SNPs
[2] Average depth of coverage calculated through Nullarbor
[3] Number of contigs > 78 bp in length

## Supplementary Table 2: Hospital A and B isolates Resistance gene profile

| Strain | Species | Date | Location | Source | ST | Plasmid | Beta-lactam resistance | | | | | Rif | Aminoglycoside resistance | | | | Quinolone resistance | | Phe | | MLS | Sul | Trim | Tet | Fos |
|---|---|---|---|---|---|---|---|---|---|---|---|---|---|---|---|---|---|---|---|---|---|---|---|---|---|
| | | | | | | | *ampC* | *bla*$_{SHV-12}$ | *bla*$_{OXA-1}$ | *bla*$_{IMP-4}$ | *bla*$_{TEM-1B}$ | *arr3* | *strB* | *strA* | *aac(6')Ib-cr* | *aac(3)-IId* | *qnrA1* | *qnrB2* | *catA2* | *catB3* | *mph(A)* | *sul1* | *dfrA18* | *tet(D)* | *fosA* |
| MS7925 | *E. coli* | 17/06/15 | A | Urine | 162 | IncHI2 | + | + | + | + | + | + | + | + | + | + | - | + | + | + | + | + | + | + | - |
| MS8078 | *E. hormaechei* | 8/11/15 | B | Swab Wound | 831 | IncHI2 | + | + | + | + | + | + | + | + | + | + | - | + | + | + | + | + | + | + | + |
| MS7923 | *E. hormaechei* | 19/05/15 | A | Rectal swab | 656 | IncHI2 | + | - | + | + | + | + | + | + | + | + | - | + | + | + | + | + | + | + | + |
| MS8077 | *E. hormaechei* | 11/11/15 | B | Urine | 830 | IncHI2 | + | - | + | + | + | + | - | - | + | + | + | + | + | + | + | + | + | + | + |
| MS8079 | *E. hormaechei* | 20/11/15 | B | Urine | 830 | IncHI2 | + | - | + | + | + | + | - | - | + | + | - | + | + | + | + | + | + | + | + |
| MS7926 | *E. hormaechei* | 11/07/15 | B | Urine | 830 | IncHI2 | + | - | + | + | + | + | - | - | + | + | + | + | + | + | + | + | + | + | + |
| MS7924 | *E. hormaechei* | 8/07/15 | A | Rectal swab | 830 | IncHI2 | + | - | - | + | + | + | - | - | + | - | - | + | + | + | + | + | + | + | + |
| MS7884[1] | *E. hormaechei* | 15/06/15 | RBWH | ETT | 90 | IncHI2 | + | - | + | + | + | + | + | + | + | + | - | + | + | + | + | + | + | + | - |

Abbreviations: Rif = rifampicin, Tet = Tetracycline, Trim = Trimethoprim, Sul = Sulphonamides, MLS = macrolide, Fos = Fosfomycin, Phe = Phenicol

[1] MS7884 – IMP+ *E. cloacae* isolated from RBWH 2015

**Supplementary Table 3: Complete chromosome and plasmid list**

| Lab number | Parent strain | Chromosome/ plasmid | Size (bp) | Isolate name | Feature |
|---|---|---|---|---|---|
| MS7969 | MS7884 (single colony) | Chromosome | 4,810,853 | MS7884B | |
| | | Plasmid | 126,208 | pMS7884B | No IMP carbapenemase gene |
| MS8407 | MS7884 (single colony grown with 2 µg/ml meropenem) | Chromosome | 4,810,853 | MS7884A | |
| | | Plasmid | 330,060 | pMS7884A | Contains multiple antibiotic resistance genes, including IMP-4 carbapenemase |

**Supplementary Table 4: Isolates cultured from environmental surveillance swabs**

| Isolate | Associated environmental sample | Cultured species ID | ST | IncHI2 plasmid? |
|---|---|---|---|---|
| M87132 | R5505 | *Klebsiella oxytoca*[1] IMP4+ | 88 | N |
| M87133 | R5506 | *Enterobacter cloacae* complex IMP4+ | 254 | Y |
| M87134 | R5514 | *Enterobacter cloacae* complex | 830 | Y |
| M87135 | R5521 | *Leclercia adecarboxylata* IMP4+ | n/a | Y |

[1] Whole genome sequencing re-identified this isolate as *Klebsiella michiganensis*

**Supplementary Table 5: MLST alleles detected in MAGs from samples with *E. hormaechei* detected based on MASH**

| MAG[1]/Isolate | *dnaA* | *fusA* | *gyrB* | *leuS* | *pyrG* | *rplB* | *rpoB* |
|---|---|---|---|---|---|---|---|
| ST90 *E. hormaechei* (MS7884) | 58 | 37 | 4 | 6 | 42 | 4 | 25 |
| R5514 | ~58 | ~37 | 62? | 6 | 42,67 | 48? | 25 |
| R5506 | - | - | - | - | - | - | 6? |
| R5522 | 129? | 8? | 21? | 41? | 15? | 79? | - |
| R5537 | 58 | ~37 | 62? | ~6 | 42,67 | 4 | 25 |

[1] MAG: metagenome-assembled genome

## Supplementary Table 6: Summary of recognition motifs[1] in MS7884A and MS7884B

| Motif string | Modification type | Methylated (%) | Number Detected | Number in Genome | Mean Score (Qmod) | Mean IPD Ratio |
|---|---|---|---|---|---|---|
| **MS7884A** | | | | | | |
| GATC | m6A | 98.2 | 50733 | 51688 | 123.40524 | 4.0679536 |
| CCYAN$_9$TGAY | m6A | 98.0 | 450 | 459 | 122.58444 | 4.87911 |
| RTCAN$_9$TRGG | m6A | 97.4 | 447 | 459 | 121.434006 | 4.6271815 |
| CAGCNAC | m6A | 97.0 | 4521 | 4662 | 112.2818 | 3.8938332 |
| **MS7884B** | | | | | | |
| GATC | m6A | 99.1 | 49412 | 49856 | 238.59088 | 4.6236324 |
| RTCAN$_9$TRGG | m6A | 99.3 | 446 | 449 | 235.08296 | 5.354307 |
| CCYAN$_9$TGAY | m6A | 99.3 | 446 | 449 | 237.71748 | 5.4802275 |
| CAGCNAC | m6A | 98.1 | 4445 | 4530 | 218.76625 | 4.31024 |
| ACCTRGCA | m6A | 55.9 | 133 | 238 | 97.1203 | 1.9882706 |

[1] Probable false positives (modified bases and motifs with a Qmod < 80) are not shown

## Supplementary Table 7: Summary of methyltransferases

| Strain | REBASE BLAST Hit[1] | Percentage identity (nt) | Query coverage | Locus Tag | Location |
|---|---|---|---|---|---|
| **MS7884A (chromosome)[2]** | | | | | |
| | M.EclNIH2 Dam GATC 813nt | 97% | 789/813 | MS7884_2116 | 2249369..2250181 |
| | M.Csa8155I GAANNNNNNntAAA 1563nt | 90% | 672/744 | MS7884_1344 | 1405871..1406629 |
| **pMS7884A (IncHI2)** | | | | | |
| | M.Sen 2050ORF235P GATC 813nt | 99% | 806/813 | MS7884_pA0268 | 227341..228153 |
| **pMS7884B (untypeable)** | | | | | |
| | MS7884_pB0001[3] | - | - | MS7884_pB0001 | 109..801 |

[1] Hits > 50 bp coverage, ≥ 90% identity with blastn against REBASE gold standard database (redundant hits excluded) downloaded 30/12/16

[2] MS7884A and MS7884B have identical gene content on the chromosome

[3] Putative methyltransferase identified on pMS7884B untypeable plasmid

## Supplementary Table 8: Primer list

| Plasmid Target | Primer Target | Product size | tm | Sequence |
|---|---|---|---|---|
| ~330 kb IncHI2 | MS7884_pA0187 *bla*IMP-4 | 200 | 58 | AGGACACACTCCAGATAACC |
| | | | 67 | TGATGCGTCTCCAGCTTCAC |
| | MS7884_pA0259 RepHI2 | 800 | 58.88 | TAATGGAGAGCGAGGGGTTC |
| | | | 58.62 | GCGGTTAAATCATGGACGGT |
| ~126 kb un-typeable | MS7884_pB0001 Putative methyltransferase | 420 | 59.21 | GAAATGTACCGCGTGCTGAA |
| | | | 58.96 | TCCTCAAGCATCTCGATCCC |
| | MS7884_pB0009 Replication initiation protein repE | 590 | 58.42 | AGAATAGCCCGCGAATTGTC |
| | | | 60.04 | CAGGAACCTACGGCGAAAGT |

# Supplementary Notes

Supplementary Note 1

1. **AmpC is unlikely to contribute to reduced carbapenem susceptibility in the 2015 RBWH isolates**

Chromosomally encoded *ampC* was detected in all 10 2015 RBWH *E. hormaechei* isolates (Table 1). While *ampC* is usually repressed and present at low levels in cells, SNPs resulting in derepression of *ampC* have been shown to increase resistance to beta-lactams [14, 15]. As such, we compared the nucleotide and amino acid sequence of our strains against several previously described SNPs in the regulatory genes *ampD*, *ampR* and *ampG* to predict *ampC* overexpression within our isolates.

All isolates contained 100% identical AmpC genes at both nucleotide and amino acid levels. The conserved AmpC residues Ser-64, Lys-67, Tyr-150, Asn-152, Lys-315 and Ala-318 [14] were all found to be present. Nucleotide and amino acid comparisons of AmpD between all 10 isolates were found to be 100% identical, with none of the previously described SNPs [16-18]. Amino acid comparison of AmpR to *E. cloacae* MNH1 [19] was 92% identical, with the highly conserved residues Arg-86, Gly-102, Ser-35, Tyr-264 and Asp-135 present in our strains. Comparison of AmpG to previous literature again found no obvious evidence to suggest heightened AmpC production [20-22]. We also checked the region upstream of *ampC* for the presence of insertion sequences (IS), as IS-driven overexpression of *ampC* has been previously reported [23-25]. Again, we found no evidence of IS upstream of *ampC*. We also found no evidence of plasmid-encoded *ampC*.

Additionally, observation of isolates grown with and without 2 ug/mL meropenem selection in LB found that colonies that lost the $bla_{\text{IMP-4}}$ carbapenemase were unable to grow under the

meropenem selective pressure, further suggesting that the *bla*$_{IMP-4}$ carbapenemase is the major driver of carbapenem resistance in these strains, and that *ampC* expression has little or no effect.

Supplementary Note 2

**2. Phenotypic observations may relate to single nucleotide variants (SNVs)**

Five core genome SNVs (4 SNPs and 1 single nucleotide deletion) differentiated the 10 RBWH *E. hormaechei* isolates (Figure 2 and Supplementary Data 1). Two isolates from Patient 3 exhibited *in vitro* phenotypic differences in adherence, suggesting that the respective SNPs may relate to within-host adaptation. Both isolates (MS7892 and MS7893) exhibited reduced pellet formation after centrifugation and resuspended more readily in solution compared to other cultures. This phenotype may have been advantageous for systemic infection, as both of these isolates were retrieved from wound and blood sites. Investigation into genotypic difference attributable to this phenotype found a non-synonymous SNP in *rcsB* (MS7884_3276) (Supplementary Data 1), a well-known gene encoding a transcriptional regulatory protein that has previously been associated with colanic acid and biofilm production, and has been shown to provide protection from the bactericidal effect of serum during bloodstream infection [12, 13]. In contrast, we note that MS7889 exhibited increased clumping and was foul smelling compared to other isolates. It encodes a nonsense mutation (GAA(E)=>TAA(STOP)) in a gene annotated as encoding SufE, a cysteine desulfuration protein (Supplementary Data 1).

Supplementary Note 3

**3. The genomes of patient 3 isolates are missing a ~25 kb phage region**

To improve our understanding of the transmission dynamics of this outbreak, we sought to identify differences in the pan-genome of these strains. Whole genome comparisons determined all 10 2015 RBWH isolates to have essentially identical genome content when compared to Ecl1. The exception was a ~25 kb region missing in all strains isolated from patient 3 (MS7890-MS7893), found to contain predicted phage-related proteins (Supplementary Figure 1). Comparison of this region to the PHAST prophage database [8] found *Escherichia* phage

HK639 (NC_016158.1) to be the closest match (35% coverage, 88% identity). This ~25 kb region represents only half of the full length of phage HK639 (~50 kb), and is present in Ecl1 and patients 1 and 2 isolates. It is unclear if the loss of this phage region could increase the fitness or virulence of the *E. hormaechei* variant infecting patient 3. Although the prophage region is likely to have been lost during the infection of patient 3, we cannot rule out that carbapenemase-producing *E. hormaechei* with and without the phage co-exist within a common environmental source in the ICU.

Supplementary Note 4

**4. PacBio complete genome sequencing identifies two plasmids carried by RBWH *E. hormaechei* isolates**

Illumina whole genome sequencing of the 10 RBWH *E. hormaechei* strains directly from clinical agar plates identified two large plasmids, although the full assemblies could not be determined using short-read data alone. Initial sequencing of isolate MS7884 (grown from a single colony) using PacBio SMRT sequencing was only able to identify one plasmid; a 126,208 bp un-typeable plasmid (pMS7884B). This plasmid lacked all of the previously identified antibiotic resistance genes determined from the Illumina sequencing data, including the $bla_{\mathrm{IMP\text{-}4}}$ carbapenemase, suggesting that the resistance plasmid had been lost during culture prior to DNA preparation. Repeat PacBio SMRT sequencing of MS7884 (grown from a single colony with 2 µg $mL^{-1}$ meropenem selection to avoid loss of $bla_{\mathrm{IMP\text{-}4}}$) enabled the successful resolution of a large, 330,060 bp IncHI2 plasmid (pMS7884A) that was found to harbour all previously identified antibiotic resistance genes. However, pMS7884B was not present in this subsequent assembly suggesting that it too had been lost during culture prior to DNA preparation. We therefore sought to determine whether these two plasmids were incompatible, and whether our clinical samples were comprised of a mixed population of carbapenemase-producing and non-producing *E. hormaechei* (See Supplementary Note 8 below).

Supplementary Note 5

**5. Summary of methylomes in complete PacBio genomes**

Sequencing two single plasmid derivatives of the same isolate with PacBio SMRT sequencing enabled us to analyse the contribution of each plasmid to the genome-wide methylation status of every nucleotide (i.e. the methylome). Supplementary Table 6 summarises the methylated motifs in MS7884A (with the ~330 kb IncHI2 plasmid) and MS7884B (with the ~126 kb untypeable plasmid), as determined using the SMRT Analysis Suite. Only one difference was determined between the two strains: an additional m6A methylated motif in MS7884B (ACCTRGCA). Searching this motif against REBASE PacBio motifs found 9 matches to previously identified PacBio motifs from *Salmonella* and *Enterobacter* species (CP015024, CP017087, CP017186, CP017187, CP017180, CP017179, CP017181, CP016012, CP016357, CP015923). As of yet an enzyme has not been determined for this motif.

To identify the causative methyltransferase (MTase) for this motif, sequences for the MS7884 chromosome, pMS7884A plasmid and pMS7884B plasmid were compared against the REBASE Gold Standard Database using BLASTn to determine MTase genes carried in the genome (Supplementary Table 7). No previously identified MTase was found in pMS7884B when compared to the Rebase Gold Standard database suggesting the presence of a novel MTase on pMS7884B. Only one putative MTase gene was annotated on the plasmid: MS7884_pB0001 – a putative DNA adenine MTase (position 109-801 bp). Searches of the Conserved Domain Database at NCBI revealed that the encoded protein matched the Pfam DNA methylase domain (pfam01555; residues 21-203, E-value = 1.21e-41) and a more specific CDD domain with a putative methylase function (PRK13699; 1-219, E-value = 4.36e-44). As this is the only annotated MTase encoded on pMS7884B it is possible that MS7884_pB0001 encodes a novel MTase that accounts for the additional methylation detected at ACCTRGCA. However, we note that methylation was relatively low frequency (55.9% of sites), the mean score and IPD ratios are also low and the motif is not typical of

adenine DNA MTases of any type, supporting the contention that MS7884_pB0001 may encode a m5C methylase that actually methylates CCWGG. Further work is required to elucidate the MTase properties (if any) of MS7884_pB0001.

Supplementary Note 6

**6. Comparison of pMS7884A and pMS7884B to published sequences**

The complete ~55kb MS7884 MDR region shares the most similarity to a region identified in pEl1573, an IncL/M plasmid carrying $bla_{IMP-4}$ (isolated in Sydney 2012), with 99% nucleotide identity across 71% of the ~55 kb MDR region (Figure 4B) [26]. pMS7884A shares 86% of its IncHI2 backbone (with 99% nucleotide identity) to the previously reported IMP-producing pEC-IMPQ (GenBank: EU855788.1) plasmid isolated from China before 2009 [27]. A carbapenemase-producing *Salmonella* sp. isolated from a domestic cat in Australia was shown to contain $bla_{IMP-4}$ within an IncHI2 MDR plasmid (pIMP4-SEM1) which is near identical to pMS7884A (Supplementary Figure 2). The most closely related plasmid to pMS7884B in the NCBI database was the IncFII plasmid pECL_A (GenBank: CP001919.1) from *E. cloacae* subsp. cloacae ATCC 13047 [28], with 64% query coverage at 99% identity (Supplementary Figure 3).

Supplementary Note 7

**7. Loss of ~34 kb MDR region in MS7889 is mediated by homologous recombination**

Compared to all other *E. hormaechei* from the 2015 RBWH isolates, MS7889 (patient 2, isolate 4) was found to have lost a number of antibiotic resistance genes, including $bla_{IMP-4}$. SMRT sequencing of the *E. hormaechei* isolate MS7884 (patient 1, isolate 1) enabled full resolution of a 330,060 bp IncHI2 plasmid harbouring a ~55 kb MDR region that encompassed the majority of antibiotic resistance genes. Analysis of this region and comparison to the draft assembly for MS7889 determined that the most likely mechanism of loss of $bla_{IMP-4}$ and other antibiotic resistance genes is homologous recombination between two near identical genes (*aacA4* and *aac(6')-Ib-cr*), resulting in the loss of a ~34 kb region (Figure 4B). Nucleotide

comparison of the two genes from MS7884 and the single *aac(6')-Ib-cr* gene from MS7889 identified only 3 SNPs between these genes (Supplementary Figure 4).

Due to the similarity between *aacA4* and *aac(6')-Ib-cr*, these genes appear as a single contig in the Illumina draft assemblies. As such, these genes have only been characterised in the complete genome for MS7884, and have been left as *aac(6')-Ib-cr* in Table 1 for the remaining Illumina draft assemblies.

Supplementary Note 8

**8. Unique SNPs in *aac(6')-Ib-cr* could contribute to increased resistance in MS7889**

The carbapenem-sensitive isolate MS7889 was found by Vitek 2 to have increased resistance to the aminoglycosides tobramycin and amikacin, despite the loss of several other antibiotic resistance genes. Comparison of amino acid translations of the aminoglycoside resistance gene *aac(6')-Ib-cr* between the SMRT sequenced MS7884 reference and the MS7889 draft assembly identified 3 non-synonymous SNPs (Supplementary Figure 5). A 329T SNP encoding Leucine was detected using MARA (7), which has previously been associated with increased amikacin resistance (28). The remaining two SNPs could further contribute to the observed increase in resistance to tobramycin and amikacin.

Supplementary Note 9

**9. Plasmid loss common during laboratory culture of *E. hormaechei* MS7884.**

Glycerol stock of the original MS7884 clinical sample was streaked onto Mueller-Hinton (MH) agar and grown overnight at 37°C. 100 colonies were pick and patched onto both plain MH agar and MH agar with 1 µg/mL meropenem and grown overnight at 37°C. 15 of 100 colonies did not grow on the meropenem plate, suggesting loss of pMS7884A or $bla_{\text{IMP-4}}$. Colony PCR determined 8 of the 15 colonies retained pMS7884A, but had lost $bla_{\text{IMP-4}}$. The remaining 7 colonies appeared to have lost pMS7884A entirely.

Colony PCR of the 100 single colonies was performed using primers designed to target pMS7884B. Of 100 colonies, only 1 appeared to have lost the pMS7884B. This confirmed that these two plasmids are not incompatible and that loss of one of the two plasmids in MS7884 is not uncommon and independent plasmid loss likely occurred prior to PacBio SMRT sequencing.

Supplementary Note 10

**10. Continued surveillance using Oxford Nanopore MinION sequencing**

Since 2015 there has been continued surveillance of *bla*$_{IMP-4}$ positive Enterobacteriaceae in RBWH. In 2017, a *bla*$_{IMP-4}$ positive *E. hormaechei* was isolated from a patient within the Hematology ward (MS14449). We used Nanopore MinION sequencing to rapidly determine whether this isolate was related to the previous ST90 *E. cloacae* outbreak. Using the MinION sequencing data alone we were able to create draft assemblies for the chromosome (~5 Mbps) and two plasmids (~382 kb and ~145 kb). However, due to a high abundance of errors we were unable to accurately determine the ST of MS14449. The larger plasmid in MS14449 appeared identical to pMS7884A based on comparison using ACT (5). The exception was a large region (position 22250-143210 in pMS7884A) that appeared to be inverted (Supplementary Figure 7). This region was flanked by IS*26* insertion sequences, which likely facilitated this inversion. The inverted region also encompasses the tetracycline and chloramphenicol resistance genes from the MDR region described in pMS7884A. Comparison of the smaller plasmid in MS14449 to pMS7884B using BRIG identified a shared core region, but large differences throughout the rest of the plasmid (Supplementary Figure 8).

In order to determine if MS14449 was clonally related to the 2015 outbreak *E. hormaechei*, we built a tree using Parsnp (v1.2) to contextualise the draft assembly for MS14449 against all other publicly available complete *E. cloacae* complex strains from NCBI (accessed 2/11/2017). This analysis placed MS14449 outside of the ST90 outbreak *E. hormaechei* cluster, indicating

that it was unlikely to be related at the strain level (Supplementary Figure 9). MS14449 was later confirmed as a different ST (ST175) using Illumina sequencing.

A *Klebsiella pneumoniae* isolate was also recovered from the same patient as MS14449 and was found to be $bla_{\text{IMP-4}}$ positive (MS14448). Illumina sequencing of this isolate and nucleotide comparison to pMS7884A indicated that it likely carries a very similar plasmid, with the exception of a small section of the MDR, which it appears to have lost (Supplementary Figure 10).

Supplementary Note 11

**11. Alternative software for single nucleotide variation (SNV) analysis**

Nesoni is a genomic toolkit developed by the Victorian Bioinformatics Consortium (https://github.com/Victorian-Bioinformatics-Consortium/nesoni) that implements read mappers and variant callers to determine SNVs (and their consequences) given sequence read datasets and a reference genome. Although in common use at the time of the initial outbreak investigation Nesoni and the SHRiMP read-mapper are no longer maintained. The original SNV calls were manually checked at the read level and were supported by observed phenotypic differences between isolates MS7889, MS7892 and MS7893 and the other *E. hormaechei* isolates, which appeared to be consistent with their respective SNV profiles (Supplementary Data 1, worksheet “Curated_SNVs” and next section of Supplementary Information).

To ensure that others can reproduce our variant calls we carried out additional read-mapping analysis with alternative software and the complete genome of *E. hormaechei* MS7884A. We found that Nesoni identified the same 4 SNVs regardless of whether SHRiMP or a recent version of Bowtie (v2.3.4.2) was implemented. We also found the same variants using the popular Snippy tool (version 4.4.0), which implements the Burrows-Wheeler Aligner (BWA) and is developed by the original author of Nesoni (https://github.com/tseemann/snippy). Finally, we note that the discrepancies between SNV calls in the MS7884A complete genome

(Supplementary Data 1) and the original SNVs presented in Figure 2 are due to reverse complementation of some sequence in the original draft reference genome of strain Ecl1 relative to the final assembly of the MS7884A chromosome.

## Supplementary Figures

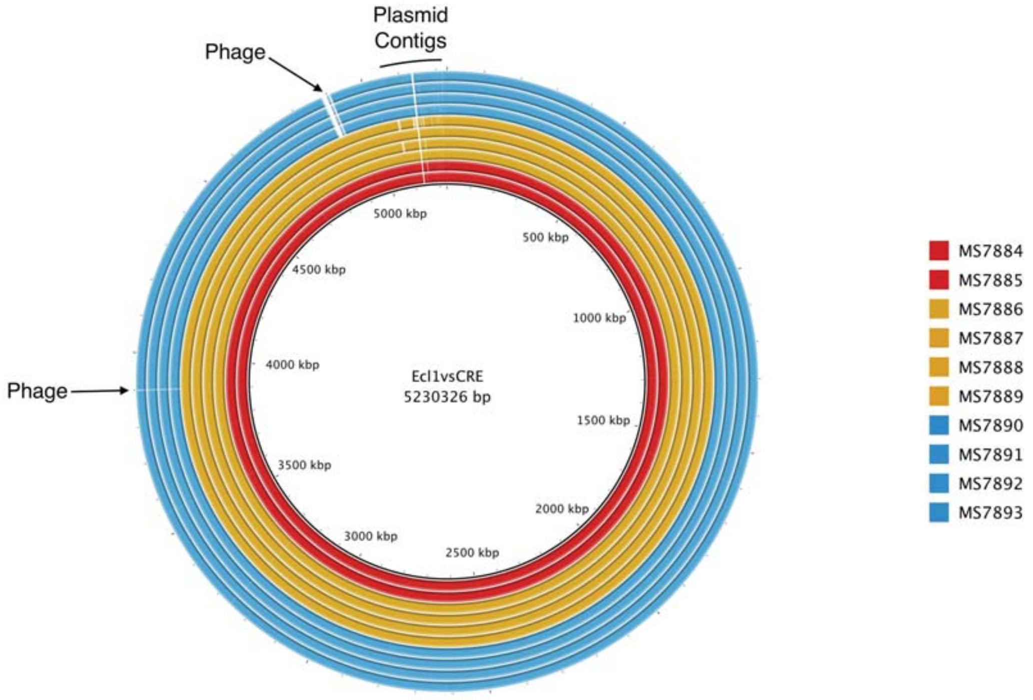


**Supplementary Figure 1: Whole genome comparison of RBWH draft assemblies:** Draft assemblies of all 2015 RBWH isolates were compared against the *E. hormaechei* Ecl1 draft reassembly (2013 RBWH, previously reported as *E. cloacae*) using BLASTn. Whole genome comparison identified two main differences between the patient 3 isolates (blue) and the patient 1 and 2 isolates (red and gold, respectively). All patient 3 isolates were missing a ~25 kb region identified as containing phage-related genes. Figure prepared using BRIG [6].

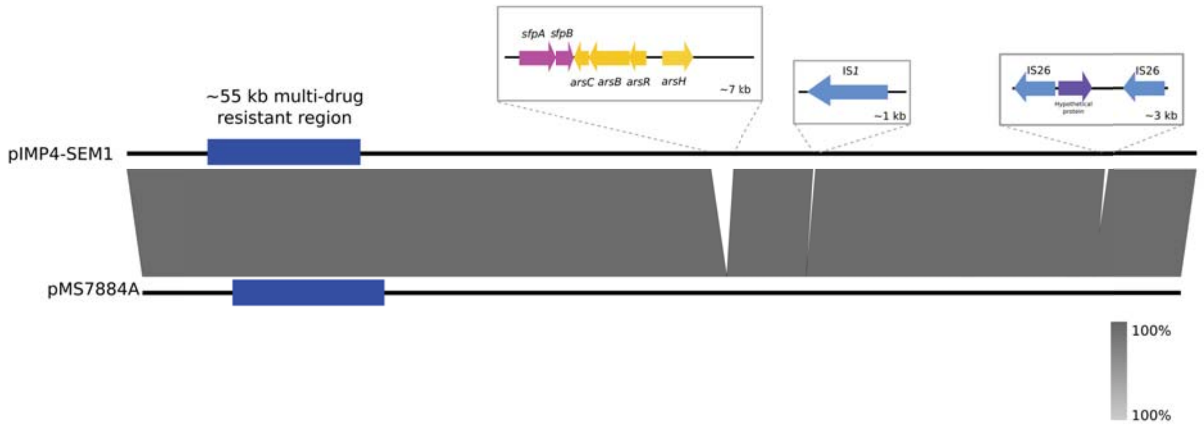


**Supplementary Figure 2: Comparison of pIMP4-SEM1 and pMS7884A:** Black lines represent the complete plasmids pIMP4-SEM1 and pMS7884A. Grey blocks indicate nucleotide identity (determined using BLASTn) between the two plasmids. Both plasmids were found to be near identical, carrying the same large ~55 kb multi-drug resistant region. Three additional regions were found in pIMP4-SEM1: a ~7 kb region carrying *sfpAB* and *arsCBRH*, an IS*1* inserted upstream of *trhR,* and an IS*26*-bound transposon carrying a hypothetical protein. Figure prepared using Easyfig.

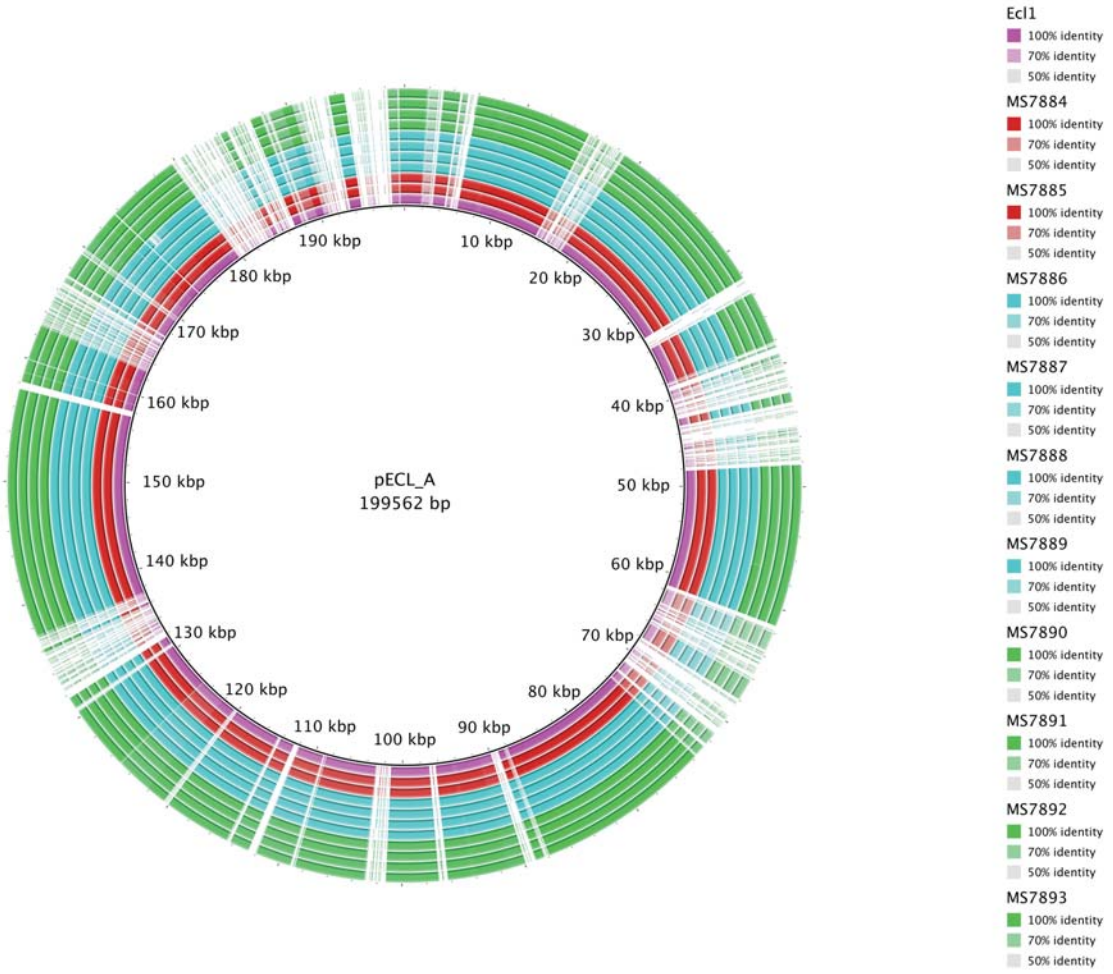


**Supplementary Figure 3: Comparison of pECL_A (GenBank: CP001919.1) from *E. cloacae* subsp. cloacae ATCC 13047 with RBWH *E. hormaechei* draft assemblies:** BLASTn comparison of pECL_A with draft assemblies of the 2013 *E. hormaechei* isolate Ecl1 (purple) and the 2015 *E. hormaechei* isolates from Patient 1 (red), Patient 2 (light blue) and Patient 3 (green). Figure prepared using BRIG [6].

```
                          1
MS7884_aacA4             ---------- ---------- ---------- ---------- -----GTGAC CAACAGCAAC
MS7889_aac(6')-lb-cr     ATGAGCAACG CAAAAACAAA GTTAGGCATC ACAAAGTACA GCATCGTGAC CAACAGCAAC
MS7884_aac(6')-lb-cr     ATGAGCAACG CAAAAACAAA GTTAGGCATC ACAAAGTACA GCATCGTGAC CAACAGCAAC

                        61
MS7884_aacA4             GATTCCGTCA CACTGCGCCT CATGACTGAG CATGACCTTG CGATGCTCTA TGAGTGGCTA
MS7889_aac(6')-lb-cr     GATTCCGTCA CACTGCGCCT CATGACTGAG CATGACCTTG CGATGCTCTA TGAGTGGCTA
MS7884_aac(6')-lb-cr     GATTCCGTCA CACTGCGCCT CATGACTGAG CATGACCTTG CGATGCTCTA TGAGTGGCTA

                       121
MS7884_aacA4             AATCGATCTC ATATCGTCGA GTGGTGGGGC GGAGAAGAAG CACGCCCGAC ACTTGCTGAC
MS7889_aac(6')-lb-cr     AATCGATCTC ATATCGTCGA GTGGTGGGGC GGAGAAGAAG CACGCCCGAC ACTTGCTGAC
MS7884_aac(6')-lb-cr     AATCGATCTC ATATCGTCGA GTGGTGGGGC GGAGAAGAAG CACGCCCGAC ACTTGCTGAC

                       181
MS7884_aacA4             GTACAGGAAC AGTACTTGCC AAGCGTTTTA GCGCAAGAGT CCGTCACTCC ATACATTGCA
MS7889_aac(6')-lb-cr     GTACAGGAAC AGTACTTGCC AAGCGTTTTA GCGCAAGAGT CCGTCACTCC ATACATTGCA
MS7884_aac(6')-lb-cr     GTACAGGAAC AGTACTTGCC AAGCGTTTTA GCGCAAGAGT CCGTCACTCC ATACATTGCA

                       241
MS7884_aacA4             ATGCTGAATG GAGAGCCGAT TGGGTATGCC CAGTCGTACG TTGCTCTTGG AAGCGGGGAC
MS7889_aac(6')-lb-cr     ATGCTGAATG GAGAGCCGAT TGGGTATGCC CAGTCGTACG TTGCTCTTGG AAGCGGGGAC
MS7884_aac(6')-lb-cr     ATGCTGAATG GAGAGCCGAT TGGGTATGCC CAGTCGTACG TTGCTCTTGG AAGCGGGGAC

                       301
MS7884_aacA4             GGATGGTGGG AAGAAGAAAC CGATCCAGGA GTACGCGGAA TAGACCAGTC ACTGGCGAAT
MS7889_aac(6')-lb-cr     GGATGGTGGG AAGAAGAAAC CGATCCAGGA GTACGCGGAA TAGACCAGTT ACTGGCGAAT
MS7884_aac(6')-lb-cr     GGAAGGTGGG AAGAAGAAAC CGATCCAGGA GTACGCGGAA TAGACCAGTT ACTGGCGAAT

                       361
MS7884_aacA4             GCATCACAAC TGGGCAAAGG CTTGGGAACC AAGCTGGTTC GAGCTCTGGT TGAGTTGCTG
MS7889_aac(6')-lb-cr     GCATCACAAC TGGGCAAAGG CTTGGGAACC AAGCTGGTTC GAGCTCTGGT TGAGTTGCTG
MS7884_aac(6')-lb-cr     GCATCACAAC TGGGCAAAGG CTTGGGAACC AAGCTGGTTC GAGCTCTGGT TGAGTTGCTG

                       421
MS7884_aacA4             TTCAATGATC CCGAGGTCAC CAAGATCCAA ACGGACCCGT CGCCGAGCAA CTTGCGAGCG
MS7889_aac(6')-lb-cr     TTCAATGATC CCGAGGTCAC CAAGATCCAA ACGGACCCGT CGCCGAGCAA CTTGCGAGCG
MS7884_aac(6')-lb-cr     TTCAATGATC CCGAGGTCAC CAAGATCCAA ACGGACCCGT CGCCGAGCAA CTTGCGAGCG

                       481
MS7884_aacA4             ATCCGATGCT ACGAGAAAGC GGGGTTTGAG AGGCAAGGTA CCGTAACCAC CCCAGATGGT
MS7889_aac(6')-lb-cr     ATCCGATGCT ACGAGAAAGC GGGGTTTGAG AGGCAAGGTA CCGTAACCAC CCCAGATGGT
MS7884_aac(6')-lb-cr     ATCCGATGCT ACGAGAAAGC GGGGTTTGAG AGGCAAGGTA CCGTAACCAC CCCATATGGT

                       541
MS7884_aacA4             CCAGCCGTGT ACATGGTTCA AACACGCCAG GCATTCGAGC GAACACGCAG TGATGCCTAA
MS7889_aac(6')-lb-cr     CCAGCCGTGT ACATGGTTCA AACACGCCAG GCATTCGAGC GAACACGCAG TGATGCCTAA
MS7884_aac(6')-lb-cr     CCAGCCGTGT ACATGGTTCA AACACGCCAG GCATTCGAGC GAACACGCAG TGATGCCTAA
```

**Supplementary Figure 4: Nucleotide alignment between MS7884 *aac(6')-Ib-cr*, MS7884 *aacA4* and MS7889 *aac(6')-Ib-cr* gene**: red boxes indicate SNP positions

```
                        1
MS7884_aacA4            ---------- -----VTNSN DSVTLRLMTE HDLAMLYEWL NRSHIVEWWG GEEARPTLAD
MS7889_aac(6')-1b-cr    MSNAKTKLGI TKYSIVTNSN DSVTLRLMTE HDLAMLYEWL NRSHIVEWWG GEEARPTLAD
MS7884_aac(6')-1b-cr    MSNAKTKLGI TKYSIVTNSN DSVTLRLMTE HDLAMLYEWL NRSHIVEWWG GEEARPTLAD

                        61                                           *                *
MS7884_aacA4            VQEQYLPSVL AQESVTPYIA MLNGEPIGYA QSYVALGSGD GWWEEETDPG VRGIDQSLAN
MS7889_aac(6')-1b-cr    VQEQYLPSVL AQESVTPYIA MLNGEPIGYA QSYVALGSGD GWWEEETDPG VRGIDQLLAN
MS7884_aac(6')-1b-cr    VQEQYLPSVL AQESVTPYIA MLNGEPIGYA QSYVALGSGD GRWEEETDPG VRGIDQLLAN

                        121                                                             *
MS7884_aacA4            ASQLGKGLGT KLVRALVELL FNDPEVTKIQ TDPSPSNLRA IRCYEKAGFE RQGTVTTPDG
MS7889_aac(6')-1b-cr    ASQLGKGLGT KLVRALVELL FNDPEVTKIQ TDPSPSNLRA IRCYEKAGFE RQGTVTTPDG
MS7884_aac(6')-1b-cr    ASQLGKGLGT KLVRALVELL FNDPEVTKIQ TDPSPSNLRA IRCYEKAGFE RQGTVTTPYG

                        181
MS7884_aacA4            PAVYMVQTRQ AFERTRSDA
MS7889_aac(6')-1b-cr    PAVYMVQTRQ AFERTRSDA
MS7884_aac(6')-1b-cr    PAVYMVQTRQ AFERTRSDA
```

**Supplementary Figure 5: Multiple sequence alignment of *aacA4* and *aac(6')-Ib-cr* amino acid sequence in MS7884 and MS7889:** red asterisks indicate amino acid change sites

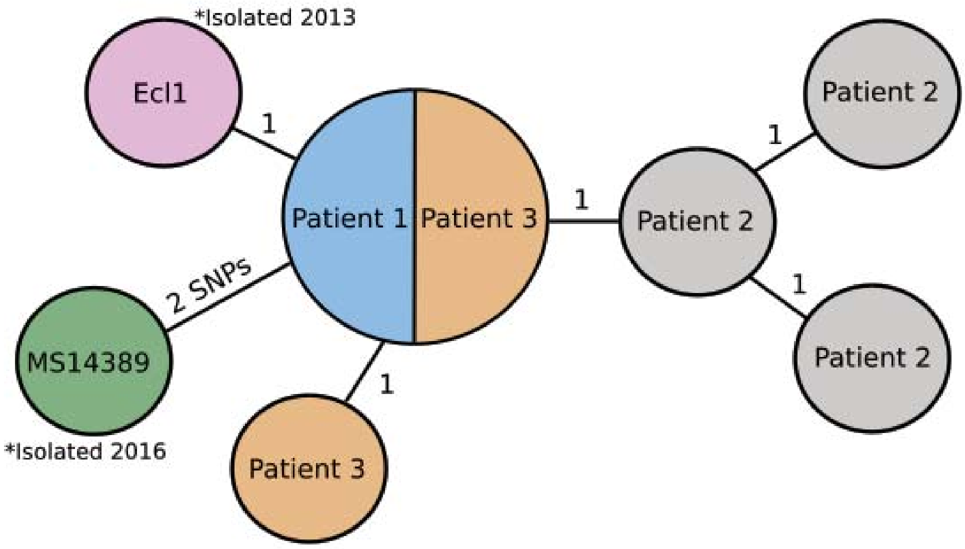


**Supplementary Figure 6: Relationship matrix of outbreak ST90 *E. hormaechei* with MS14389**

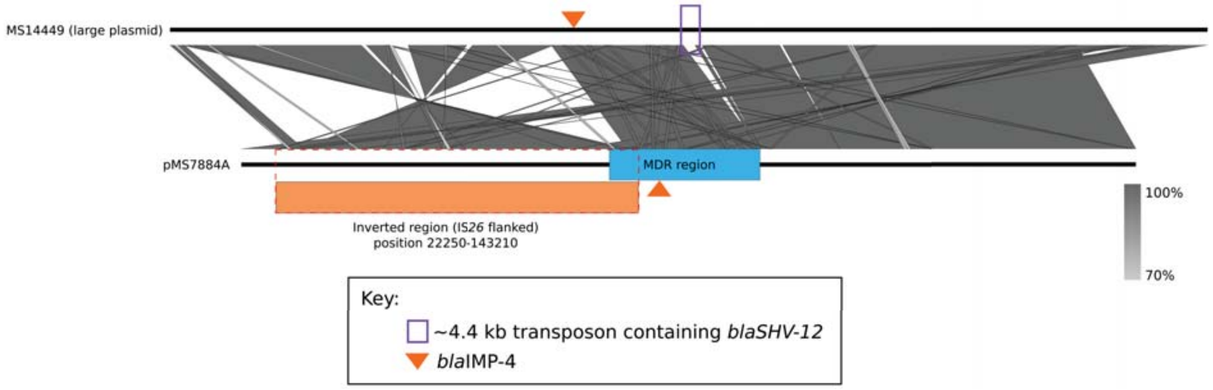


**Supplementary Figure 7: Comparison of large plasmid from MS14449 and pMS7884A:**

BLASTn comparison shown in grey. Figure prepared using Easyfig.

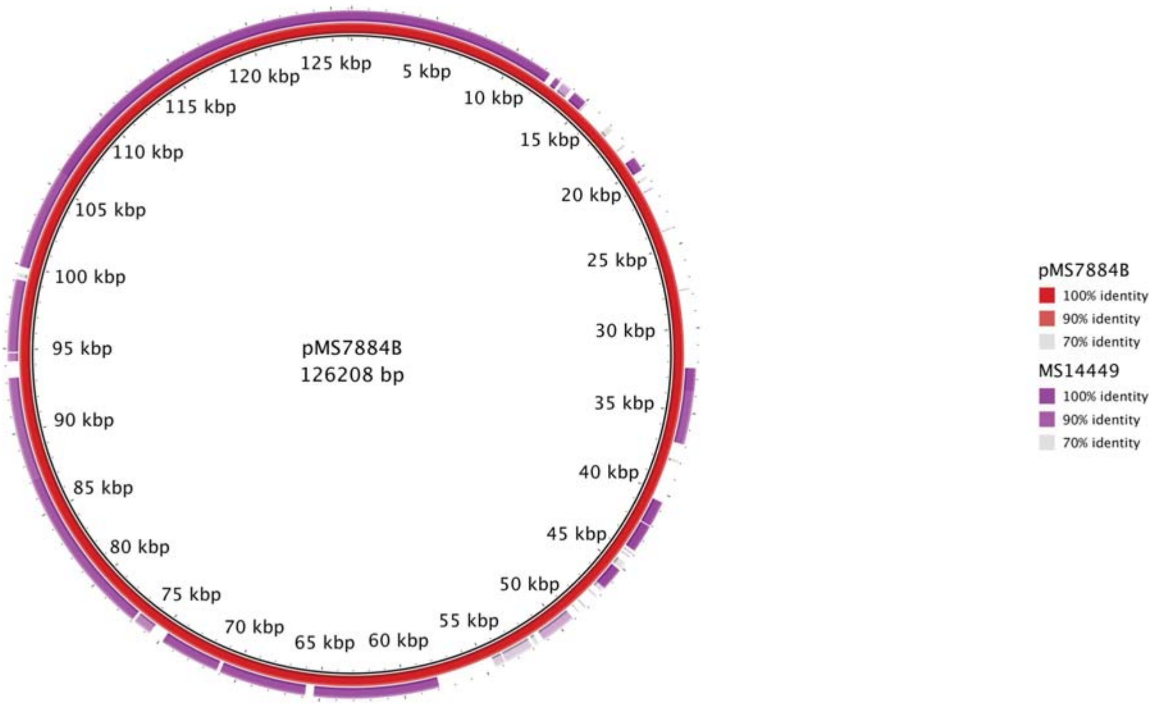


**Supplementary Figure 8: Comparison of pMS7884B and small plasmid from MS14449:** BLASTn comparison between pMS788B with itself (red) and small plasmid from MS14449 (purple). Figure prepared using BRIG.

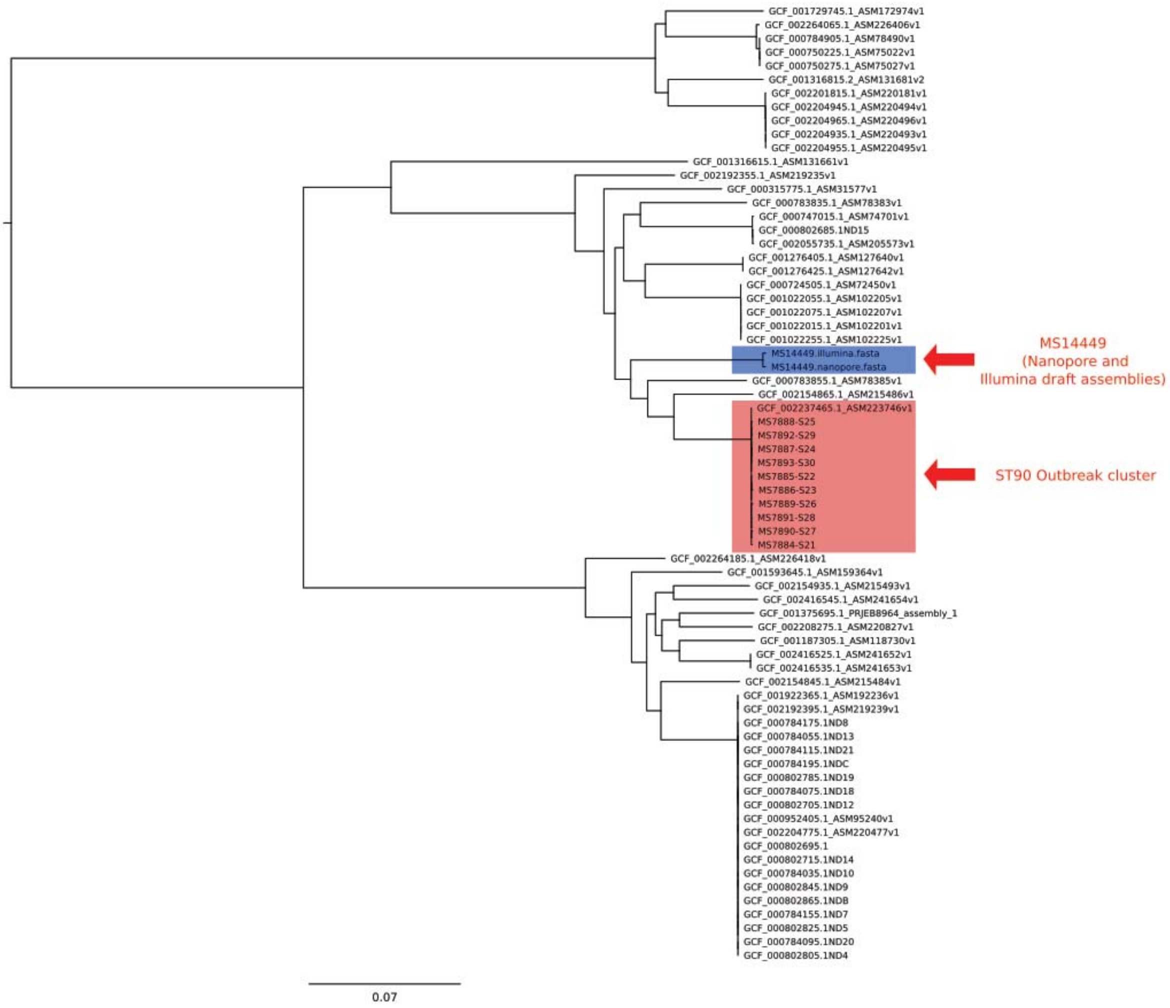


**Supplementary Figure 9: Parsnp tree contextualising MS14449 Illumina and Nanopore draft assemblies against ST90 outbreak cluster and other complete publicly available *E. cloacae* complex strains**

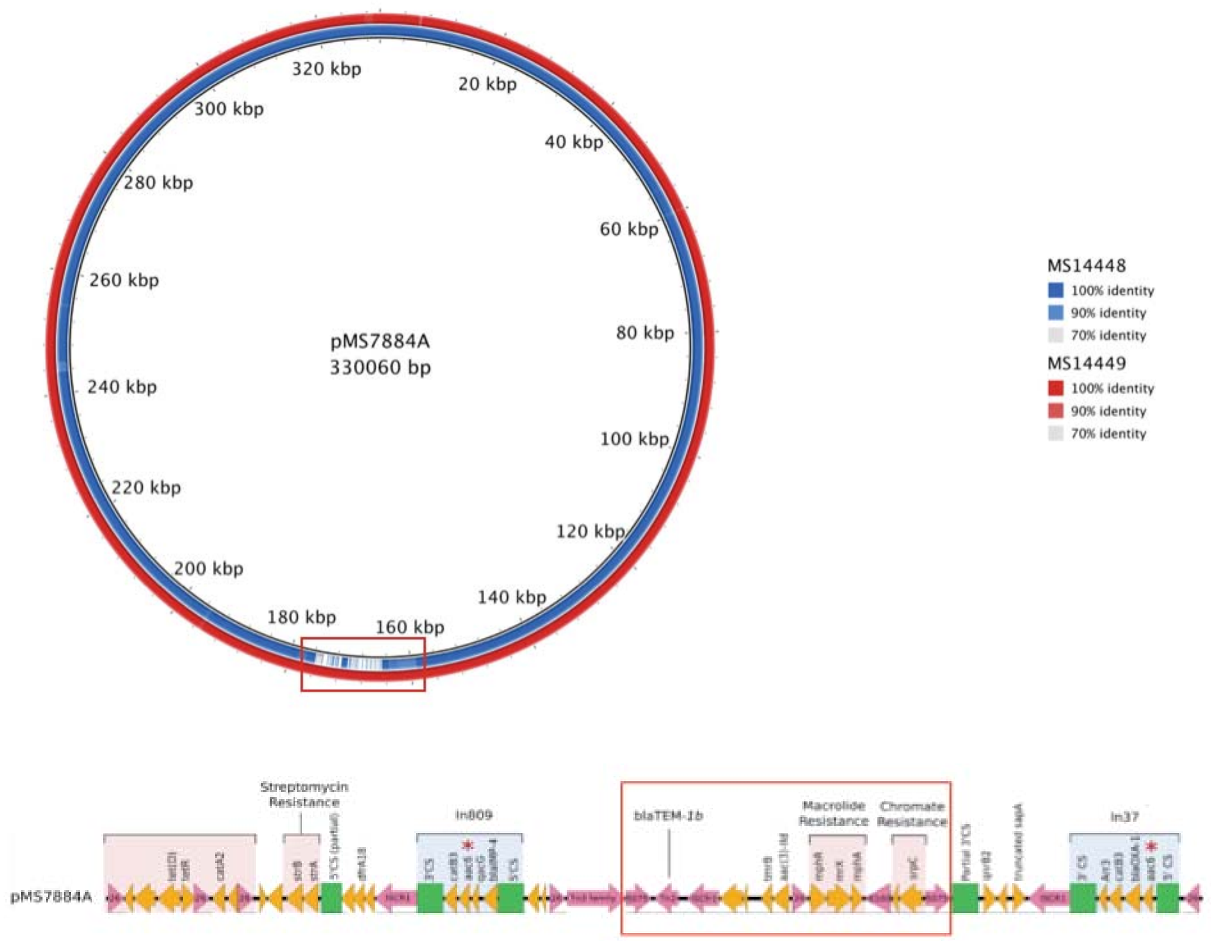


**Supplementary Figure 10: Comparison of pMS7884A with MS14448 and MS14449 draft assemblies:** BLASTn comparison between pMS788A with *K. pneumoniae* MS14448 (blue) and *E. hormaechei* MS14449 (red). Red box indicates region missing from *K. pneumoniae* MS14448 IncHI2 plasmid. Figure prepared using BRIG and Easyfig.

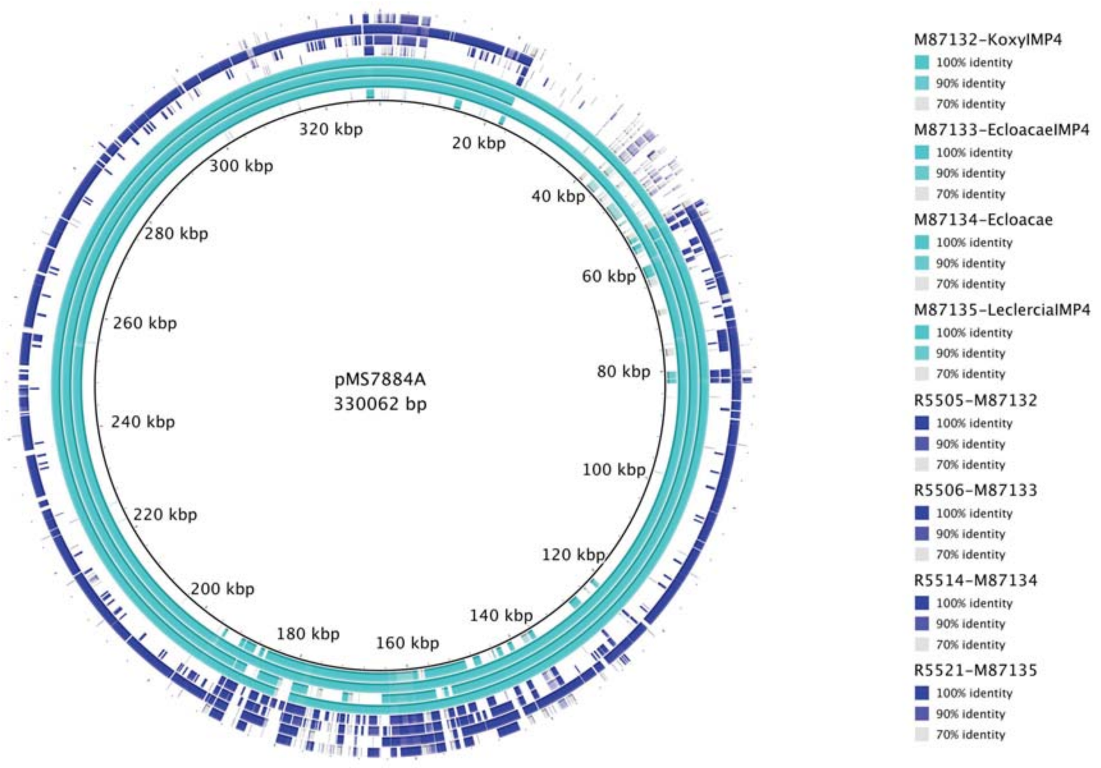


**Supplementary Figure 11: Comparison of isolate draft assemblies and associated MAGs from metagenomic sequencing of associated environmental samples (dark blue) against IncHI2 plasmid pMS7884A:** BLASTn comparisons between pMS7884A and isolates (light blue, see Supplementary Table 4) and environmental metagenomic samples (dark blue) are shown from inner-to-outer ring according to the top-to-bottom order on the right hand side legend. Figure prepared using BRIG.

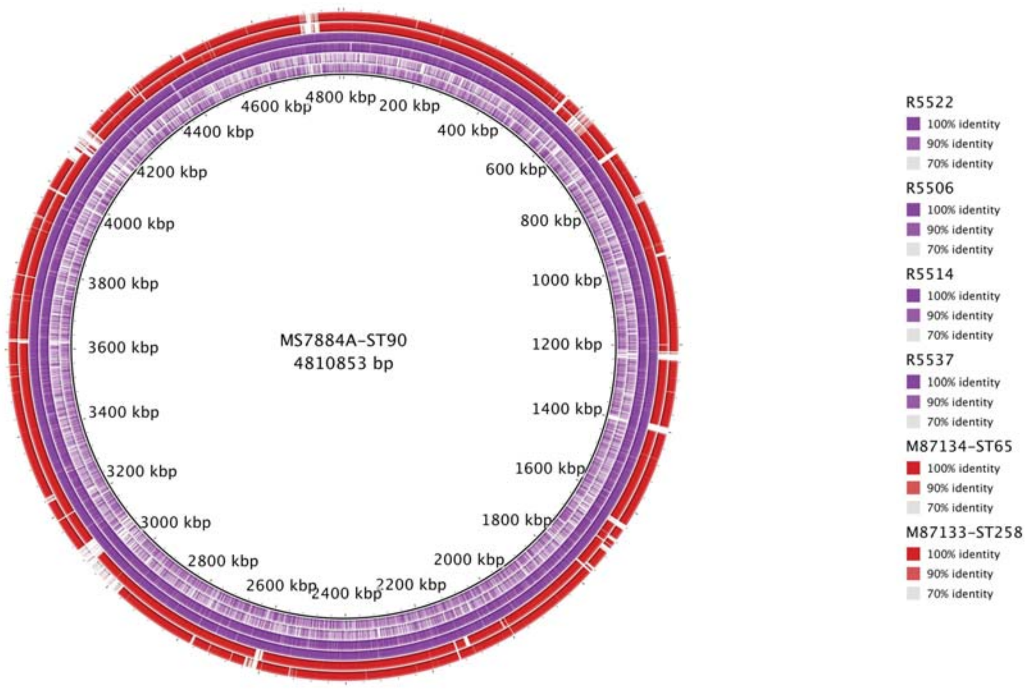


**Supplementary Figure 12: Comparison of ST90 *E. hormaechei* strain MS7884A with MAGs from samples with positive *E. hormaechei*:** BLASTn comparisons between MS7884A with environmental metagenomic samples (purple; positive for *E. hormaechei* as determined by MASH) and two non-ST90 *E. cloacae* complex isolates (red, see supplementary Table 4) are shown from inner-to-outer ring according to the top-to-bottom order on the right hand side legend. R5514 and R5537 appear to have high identity to MS7884A. Figure prepared using BRIG.

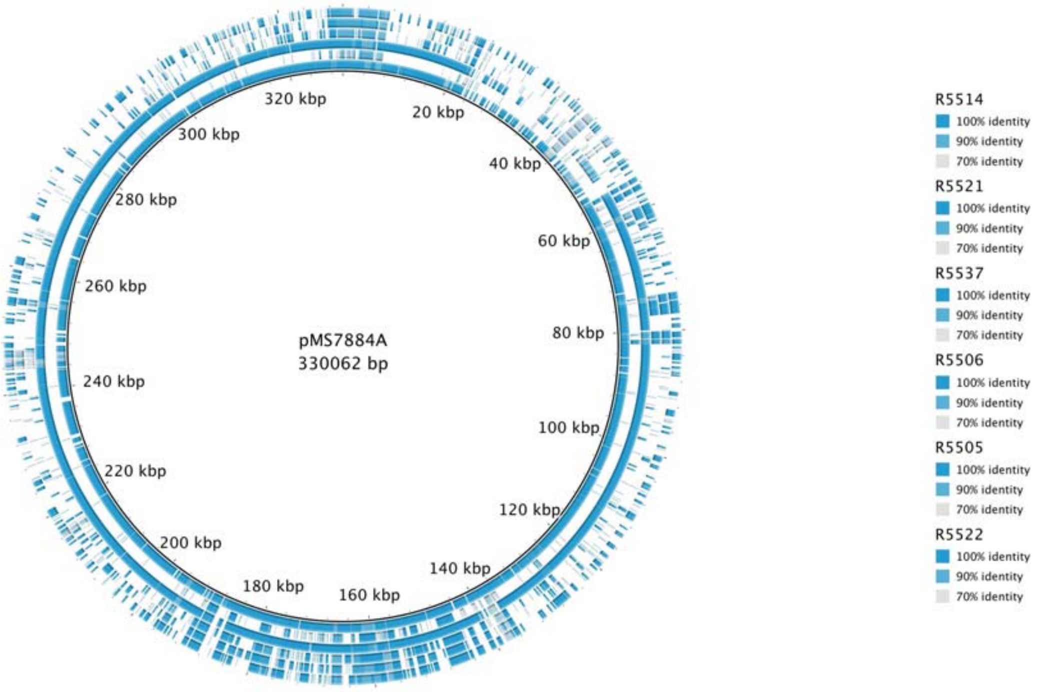


**Supplementary Figure 13: Comparison of the IncHI2 plasmid pMS7884 with MAGs from samples with positive pMS7884A and/or the associated MDR region:** BLASTn comparisons between pMS7884A and environmental metagenomic samples are shown from inner-to-outer ring according to the top-to-bottom order on the right hand side. Two samples (R5514 and R5537) appear to have high identity to pMS7884A. Figure prepared using BRIG.

# Supplementary References


1. Victorian-Bioinformatics-Consortium. *Nesoni*. 2014 [cited 2016; v 0.130:Available from: https://github.com/Victorian-Bioinformatics-Consortium/nesoni.
2. Ondov, B.D., et al., *Mash: fast genome and metagenome distance estimation using MinHash.* Genome Biol, 2016. **17**(1): p. 132.
3. Jain, C., et al., *High throughput ANI analysis of 90K prokaryotic genomes reveals clear species boundaries.* Nat Commun, 2018. **9**(1): p. 5114.
4. Sidjabat, H.E., et al., *Interspecies transfer of blaIMP-4 in a patient with prolonged colonization by IMP-4-producing Enterobacteriaceae.* J Clin Microbiol, 2014. **52**(10): p. 3816-8.
5. Bankevich, A., et al., *SPAdes: A New Genome Assembly Algorithm and Its Applications to Single-Cell Sequencing.* Journal of Computational Biology, 2012. **19**(5): p. 455-477.
6. Alikhan, N.F., et al., *BLAST Ring Image Generator (BRIG): simple prokaryote genome comparisons.* BMC Genomics, 2011. **12**: p. 402.
7. Carver, T.J., et al., *ACT: the Artemis Comparison Tool.* Bioinformatics, 2005. **21**(16): p. 3422-3.
8. Zhou, Y., et al., *PHAST: a fast phage search tool.* Nucleic Acids Res, 2011. **39**(Web Server issue): p. W347-52.
9. Tsafnat, G., J. Copty, and S.R. Partridge, *RAC: Repository of Antibiotic resistance Cassettes.* Database, 2011. **2011**(0): p. bar054-bar054.
10. Moura, A., et al., *INTEGRALL: a database and search engine for integrons, integrases and gene cassettes.* Bioinformatics, 2009. **25**(8): p. 1096-8.
11. Varani, A.M., et al., *ISsaga is an ensemble of web-based methods for high throughput identification and semi-automatic annotation of insertion sequences in prokaryotic genomes.* Genome Biology, 2011. **12**(3): p. R30.
12. Miajlovic, H., et al., *Response of Extraintestinal Pathogenic Escherichia coli to Human Serum Reveals a Protective Role for Rcs-Regulated Exopolysaccharide Colanic Acid.* Infection and Immunity, 2014. **82**(1): p. 298-305.
13. Lacour, S., et al., *Tyrosine Phosphorylation of the UDP-Glucose Dehydrogenase of Escherichia coli Is at the Crossroads of Colanic Acid Synthesis and Polymyxin Resistance.* PLOS ONE, 2008. **3**(8): p. e3053.
14. Jacoby, G.A., *AmpC beta-lactamases.* Clin Microbiol Rev, 2009. **22**(1): p. 161-82, Table of Contents.
15. Babouee Flury, B., et al., *Association of Novel Nonsynonymous Single Nucleotide Polymorphisms in ampD with Cephalosporin Resistance and Phylogenetic Variations in ampC, ampR, ompF, and ompC in Enterobacter cloacae Isolates That Are Highly Resistant to Carbapenems.* Antimicrob Agents Chemother, 2016. **60**(4): p. 2383-90.
16. Stapleton, P., K. Shannon, and I. Phillips, *DNA sequence differences of ampD mutants of Citrobacter freundii.* Antimicrob Agents Chemother, 1995. **39**(11): p. 2494-8.
17. Kopp, U., et al., *Sequences of wild-type and mutant ampD genes of Citrobacter freundii and Enterobacter cloacae.* Antimicrob Agents Chemother, 1993. **37**(2): p. 224-8.
18. Ehrhardt, A.F., et al., *Sequencing and analysis of four new Enterobacter ampD Alleles.* Antimicrob Agents Chemother, 1996. **40**(8): p. 1953-6.
19. Kuga, A., R. Okamoto, and M. Inoue, *ampR gene mutations that greatly increase class C beta-lactamase activity in Enterobacter cloacae.* Antimicrob Agents Chemother, 2000. **44**(3): p. 561-7.
20. Korfmann, G. and C.C. Sanders, *ampG is essential for high-level expression of AmpC beta-lactamase in Enterobacter cloacae.* Antimicrob Agents Chemother, 1989. **33**(11): p. 1946-51.

21. Lindquist, S., et al., *AmpG, a signal transducer in chromosomal beta-lactamase induction.* Mol Microbiol, 1993. **9**(4): p. 703-15.
22. Zhang, Y., et al., *ampG gene of Pseudomonas aeruginosa and its role in beta-lactamase expression.* Antimicrob Agents Chemother, 2010. **54**(11): p. 4772-9.
23. Bagge, N., et al., *Constitutive high expression of chromosomal beta-lactamase in Pseudomonas aeruginosa caused by a new insertion sequence (IS1669) located in ampD.* Antimicrob Agents Chemother, 2002. **46**(11): p. 3406-11.
24. Lindberg, F., S. Lindquist, and S. Normark, *Inactivation of the ampD gene causes semiconstitutive overproduction of the inducible Citrobacter freundii beta-lactamase.* J Bacteriol, 1987. **169**(5): p. 1923-8.
25. Hilty, M., et al., *Characterisation and clinical features of Enterobacter cloacae bloodstream infections occurring at a tertiary care university hospital in Switzerland: is cefepime adequate therapy?* Int J Antimicrob Agents, 2013. **41**(3): p. 236-49.
26. Partridge, S.R., et al., *pEl1573 Carrying blaIMP-4, from Sydney, Australia, is closely related to other IncL/M plasmids.* Antimicrob Agents Chemother, 2012. **56**(11): p. 6029-32.
27. Chen, Y.T., et al., *Mobilization of qnrB2 and ISCR1 in plasmids.* Antimicrob Agents Chemother, 2009. **53**(3): p. 1235-7.
28. Ren, Y., et al., *Complete genome sequence of Enterobacter cloacae subsp. cloacae type strain ATCC 13047.* J Bacteriol, 2010. **192**(9): p. 2463-4.
