# Supplementary material for: Integrating multiple genomic technologies to investigate an outbreak of carbapenemase-producing Enterobacter hormaechei
Source: Nat Commun. 2020 Jan 24;11:466. doi: 10.1038/s41467-019-14139-5 (PMC6981164; doi:10.1038/s41467-019-14139-5)
Supplement: Supplementary file 3 — Description of Additional Supplementary Files [file 41467_2019_14139_MOESM3_ESM.pdf]

Description of Additional Supplementary Files

File Name: Supplementary Data 1

Description: **Raw and curated variant calls.** Raw SNVs worksheet contains variant calls from 2015 RBWH *E. hormaechei* Illumina genome data mapped to the original or reassembled version of *E. hormaechei* Ecl1 draft genome assembly as reference. Curated SNVs worksheet contains only the high quality manually curated variant calls and consequences, along with their coordinates in the MS7884 complete genome.

File Name: Supplementary Data 2

Description: **Environmental metagenomic analysis.** Contains read-mapping, assembly and FastANI results for environmental metagenomic sequence datasets
