# Supplementary material for: Integrating multiple genomic technologies to investigate an outbreak of carbapenemase-producing Enterobacter hormaechei
Source: Nat Commun. 2020 Jan 24;11:466. doi: 10.1038/s41467-019-14139-5 (PMC6981164; doi:10.1038/s41467-019-14139-5)
Supplement: Supplementary file 6 — Reporting Summary [file 41467_2019_14139_MOESM6_ESM.pdf]

nature research

Corresponding author(s): Scott Beatson

Last updated by author(s): Nov 25, 2019

Reporting Summary

Nature Research wishes to improve the reproducibility of the work that we publish. This form provides structure for consistency and transparency in reporting. For further information on Nature Research policies, see Authors & Referees and the Editorial Policy Checklist.

Statistics

For all statistical analyses, confirm that the following items are present in the figure legend, table legend, main text, or Methods section.

n/a	Confirmed	
☒	☐	The exact sample size (*n*) for each experimental group/condition, given as a discrete number and unit of measurement
☒	☐	A statement on whether measurements were taken from distinct samples or whether the same sample was measured repeatedly
☒	☐	The statistical test(s) used AND whether they are one- or two-sided<br>*Only common tests should be described solely by name; describe more complex techniques in the Methods section.*
☒	☐	A description of all covariates tested
☒	☐	A description of any assumptions or corrections, such as tests of normality and adjustment for multiple comparisons
☒	☐	A full description of the statistical parameters including central tendency (e.g. means) or other basic estimates (e.g. regression coefficient) AND variation (e.g. standard deviation) or associated estimates of uncertainty (e.g. confidence intervals)
☒	☐	For null hypothesis testing, the test statistic (e.g. *F*, *t*, *r*) with confidence intervals, effect sizes, degrees of freedom and *P* value noted<br>*Give P values as exact values whenever suitable.*
☒	☐	For Bayesian analysis, information on the choice of priors and Markov chain Monte Carlo settings
☒	☐	For hierarchical and complex designs, identification of the appropriate level for tests and full reporting of outcomes
☒	☐	Estimates of effect sizes (e.g. Cohen's *d*, Pearson's *r*), indicating how they were calculated

Our web collection on statistics for biologists contains articles on many of the points above.

Software and code

Policy information about availability of computer code

Data collection	Not applicable.
Data analysis	Illumina sequence data was assembled with Spades v3.6.0. Assembly metrics were checked with QUAST v2.3. Kraken v0.10.5-beta and FastANI v1.1 were used for taxonomic assignment. Readmapping and variant calling was carried out with SHRiMP v2.2.3 or Bowtie v2.3.4.2 as implemented in Nesoni v0.130, or snippy 4.4.0. RAxML v8.1.15 was used for maximum likelihood phylogenetic reconstruction. In silico MLST used srst2 v0.1.5, Similarity searches were performed with BLASTn v2.2.3. MASH v1.1.1, Samtools fastq (v1.9), Spades v3.11.1, Abricate v0.8 were used for metagenomic analyses.

For manuscripts utilizing custom algorithms or software that are central to the research but not yet described in published literature, software must be made available to editors/reviewers. We strongly encourage code deposition in a community repository (e.g. GitHub). See the Nature Research guidelines for submitting code & software for further information.

Data

Policy information about availability of data

All manuscripts must include a data availability statement. This statement should provide the following information, where applicable:

- Accession codes, unique identifiers, or web links for publicly available datasets
- A list of figures that have associated raw data
- A description of any restrictions on data availability

Genome data has been deposited under Bioproject PRJNA383436 [https://www.ncbi.nlm.nih.gov/bioproject/PRJNA383436] . Illumina sequence read data has been deposited to the sequence read archive (SRA) under the accessions SRR5821451 – SRR5821467, SRR8789021 and SRR8789023-SRR8789027 [https://trace.ncbi.nlm.nih.gov/Traces/sra/?study=SRP106560]. Nanopore sequence read data has been deposited to the SRA under the accession SRR8789022 [https://trace.ncbi.nlm.nih.gov/Traces/sra/?run=SRR8789022], PacBio sequence read data has been deposited to the SRA under the accessions SRR5821468 [https://trace.ncbi.nlm.nih.gov/Traces/sra/?run=SRR5821468] and SRR5821469 [https://trace.ncbi.nlm.nih.gov/Traces/sra/?run=SRR5821469]. Environmental metagenomic sequence read data has been deposited to the SRA under the accessions SRR8801892-SRR8801897 [https://trace.ncbi.nlm.nih.gov/Traces/study/?

acc=SRP106560&o=acc_s%3Aa] . The complete genome of MS7884 has been deposited to Genbank under the accessions CP022532-CP022534 [https://www.ncbi.nlm.nih.gov/assembly/GCA_002237465.1].

Field-specific reporting

Please select the one below that is the best fit for your research. If you are not sure, read the appropriate sections before making your selection.

☒ Life sciences ☐ Behavioural & social sciences ☐ Ecological, evolutionary & environmental sciences

For a reference copy of the document with all sections, see nature.com/documents/nr-reporting-summary-flat.pdf

Life sciences study design

All studies must disclose on these points even when the disclosure is negative.

Sample size	Sample sizes were determined based on availability of isolates for sequence analysis
Data exclusions	Not applicable.
Replication	This study involved the collection and genome sequencing of E. hormaechei isolates from patients admitted to QLD hospitals in 2015-2017. It also involved the collection of environmental samples from a Brisbane hospital in 2018. It would not be possible to repeat these experiments using a controlled protocol.
Randomization	Randomization was not relevant to this study
Blinding	Blinding was not relevant to this study.

Reporting for specific materials, systems and methods

We require information from authors about some types of materials, experimental systems and methods used in many studies. Here, indicate whether each material, system or method listed is relevant to your study. If you are not sure if a list item applies to your research, read the appropriate section before selecting a response.

Materials & experimental systems

n/a	Involved in the study	
☒	☐	Antibodies
☒	☐	Eukaryotic cell lines
☒	☐	Palaeontology
☒	☐	Animals and other organisms
☐	☒	Human research participants
☒	☐	Clinical data

Methods

n/a	Involved in the study	
☒	☐	ChIP-seq
☒	☐	Flow cytometry
☒	☐	MRI-based neuroimaging

Human research participants

Policy information about studies involving human research participants

Population characteristics	Two patients (patient 1: female age 43, patient 2: female age 58) in mid 2015 were transferred from regional Queensland hospitals to the ICU with burn injuries sustained from the same accident. Patient 3, a 39-year old woman, was admitted with 66% total body surface area burns to the same ICU 5 weeks after the first two patients.
Recruitment	E. hormaechei isolates were collected from these three patients and characterised using whole genome sequencing.
Ethics oversight	Ethical approval for this work was provided by the Metro North Hospital and Health Service Human Research Ethics Committee (HREC/16/QRBW/253). In addition, written consent was obtained from the patients or their next of kin to publish clinical details.

Note that full information on the approval of the study protocol must also be provided in the manuscript.
